# Efficacy of pulsed ultraviolet (PUV) light for disinfection of nosocomial pathogens: An in-vitro investigation of key parameters for surface and equipment applications

**DOI:** 10.1371/journal.pone.0338944

**Published:** 2025-12-16

**Authors:** Mohammad Kazem Sharifi-Yazdi, Sina Sharifi-Yazdi, Sara Sharifi Yazdi

**Affiliations:** Tehran University of Medical Sciences, Tehran, Iran; Satyawati College, University of Delhi, INDIA

## Abstract

Hospital-acquired infections remain a persistent challenge, particularly when caused by bacterial strains that have developed resistance to multiple antibiotics. Reports from both clinical wards and public health agencies show how rapidly these organisms adapt, leaving many standard cleaning procedures and treatments far less effective than they once were. This concern prompted us to investigate pulsed ultraviolet (PUV) light from a xenon source as an alternative approach to inactivation, offering rapid action without the use of harsh chemicals. The work involved testing four bacterial species of clinical relevance: *Pseudomonas aeruginosa*, *Staphylococcus aureus*, *Bacillus cereus*, and spores of *Bacillus megaterium*. We evaluated the impact of UV dose, examined how suspension depth influenced performance, and assessed whether exposure to visible light after treatment could reverse the effect. For all strains, significant microbial reduction was achieved using a broad-spectrum emission with a strong germicidal peak in the 260 nm region. Spores showed much greater resilience than vegetative cells, while increased liquid depth reduced the disinfection efficiency.Some degree of photorepair occurred in non-spore-forming species under standard room lighting. Taken together, these findings indicate that, when tuned to the right parameters, PUV could serve as a valuable addition to hospital disinfection routines, especially for equipment and surfaces that cannot withstand heat or aggressive chemical agents.

## 1. Introduction

Nosocomial infections account for hundreds of thousands of deaths worldwide annually [1]. *Pseudomonas aeruginosa* alone is responsible for 5–15% of infections in European ICUs [2], and methicillin-resistant *Staphylococcus aureus* (MRSA) is a leading cause of mortality in the United States [3]. The rapid evolution of resistance in these bacteria has rendered many conventional chemical disinfectants less effective [4], necessitating the search for alternative disinfection methods.

Pulsed ultraviolet (PUV) technology offers a chemical-free, clean option by creating pyrimidine dimers in microbial DNA, thereby halting replication [5]. Unlike traditional continuous-wave UV units, which typically use low-pressure mercury lamps emitting at a single wavelength (254 nm), PUV systems utilize xenon flashlamps to deliver high-intensity,broad-spectrum pulses. This high-peak-power delivery enhances microbial inactivation, potentially overwhelming cellular repair mechanisms more effectively than continuous exposure [6]. Studies have demonstrated that UV-C irradiation exhibits potent antimicrobial effects against various pathogenic bacteria, including clinically significant isolates of *Pseudomonas aeruginosa* [7]. This study aims to ascertain the efficacy of PUV against key nosocomial pathogens. Key parameters such as dose-response kinetics, the effect of suspension depth, and the potential for photorepair on disinfection effectiveness are studied systematically.

## 2. Materials and methods

### 2.1. Bacterial strains and culture conditions

Four reference and clinical strains were utilized: *Pseudomonas aeruginosa* (NCTC 10662), *Staphylococcus aureus* (Clinical isolate, Glasgow Royal Infirmary), *Bacillus cereus* (NCTC 2599), and *Bacillus megaterium* (NCTC 10342). Cultures were maintained on Tryptic Soy Agar (TSA, Merck, Germany) at 37°C with 24-hour subculturing. Methodological approaches were designed to be consistent with standard laboratory practices for UV disinfection assays [8].

#### 2.1.1. Spore preparation and verification.

To obtain spores, *B. megaterium* was cultivated on Nutrient Agar (Merck,Germany) plates for 7 days at 30°C to induce sporulation. Spores were harvested by washing the agar surface with sterile distilled water. The suspension was washed three times by centrifugation (5000 x g for 10 min) and resuspension in sterile distilled water to remove vegetative debris. The suspension was then heat-treated at 80°C for 15 minutes to eliminate any remaining vegetative cells. Spore formation (>95%) was verified under phase-contrast microscopy before use. The final spore concentration was adjusted to approximately 10⁸ spores/mL.

### 2.2. PUV irradiation system

**Fig 1:**
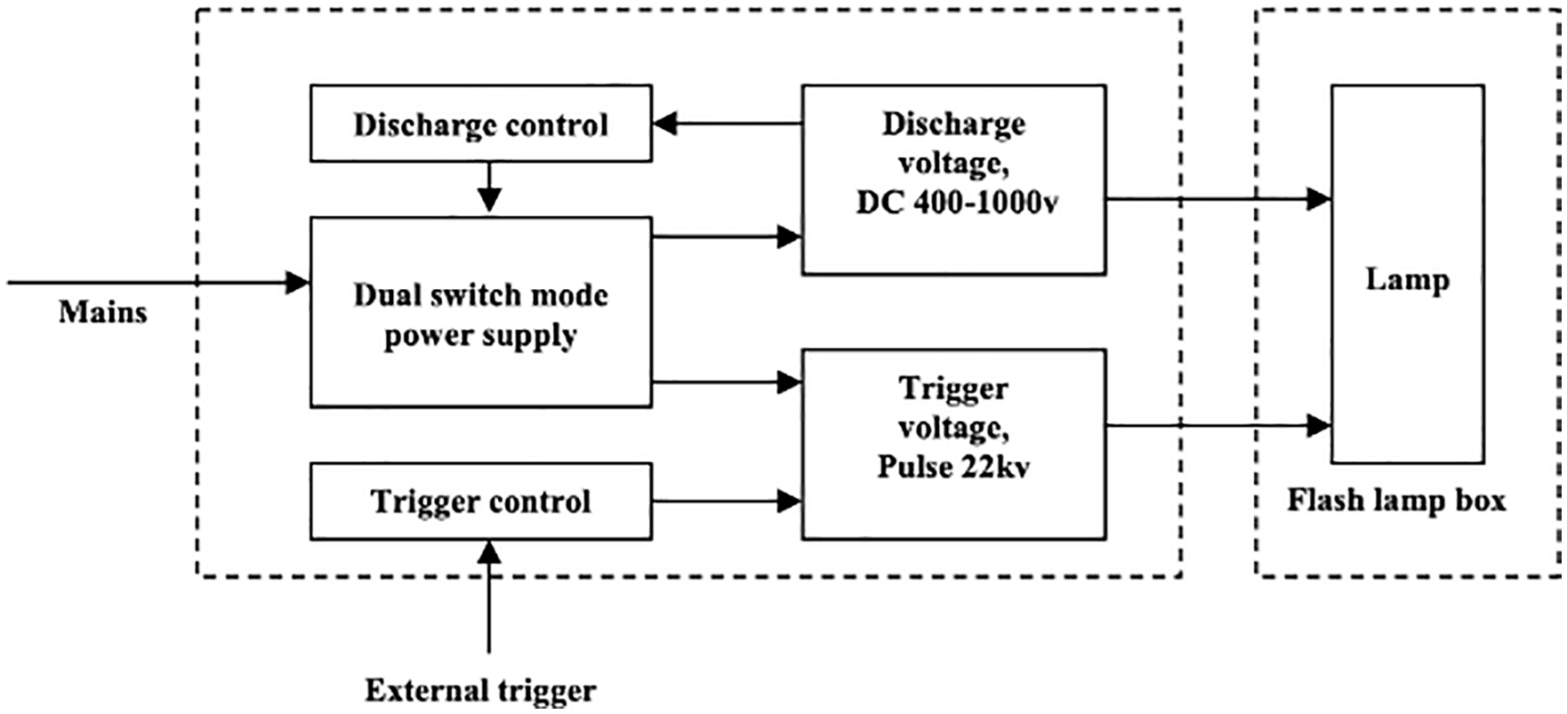
Block diagram of the pulsed UV system (Adapted from the manufacturer’s manual).

Experiments utilized a SAMTECH PUV-01 system (Samtech, UK), which employs a low-pressure (450 torr) xenon flash lamp. The system generates intense, broad-spectrum UV light, with significant output in the germicidal UVC range (220–280 nm). Fig 1 shows a block diagram of the system setup.

A standard polystyrene Petri dish containing 20 ml of the test sample was placed at the bottom of a UV-shielded enclosure. The flash lamp was positioned 8 cm above the sample surface. For all experiments, the pulsed UV source was operated at a charging voltage of 1 kV, delivering 20 J of electrical energy per pulse, with a frequency of 1 pulse/s. The flash lamp was preheated for 5 min prior to UV exposures to obtain a reproducible UV output. The applied UV dose was controlled by the number of pulses.

#### 2.2.1. UV dose measurement.

The UV dose (fluence) was determined based on the manufacturer’s specifications for the SAMTECH PUV-01 system. According to the calibration data, at a distance of 10 cm and a discharge voltage of 1000V, the system delivers a fluence of 346 µJ/cm² per pulse in the < 300 nm wavelength band. As our experiments were conducted at a closer distance of 8 cm, the actual germicidal fluence at the sample surface is estimated at approximately **540 µJ/cm**^**2**^ **(0.54 mJ/cm**^**2**^**)** per pulse based on the inverse square law. This value was used to calculate the cumulative dose for dose-response analyses.

#### 2.2.2. Safety precautions.

All procedures were conducted with strict adherence to safety guidelines. The high-voltage components and intense UV radiation from the system are potentially lethal. All experiments were performed within a shielded, interlocked enclosure to prevent accidental exposure. Operators used appropriate personal protective equipment (UV-blocking face shields, gloves, and lab coats) at all times.

### 2.3. Experimental procedures

All experiments were conducted in triplicate (n = 3). Dark controls (no-UV exposure) were included for all experiments, and no significant reduction in viable counts was observed. Samples were gently agitated prior to exposure to ensure a homogenous suspension.

#### 2.3.1. Dose-response analysis.

Vegetative cells and spores (10⁸ CFU/mL) in Phosphate-Buffered Saline (PBS, pH 7.4) were exposed to an increasing number of pulses (from 0 to 300). Survival was quantified by standard plate count on TSA after 24–48 hours of incubation at 37°C. Survival curves were plotted to determine inactivation kinetics.

#### 2.3.2. Suspension depth effect.

Suspensions of *S. aureus* (10⁸ CFU/mL) in volumes of 20, 30, 40, and 50 mL (corresponding to different depths in the Petri dish) were exposed to a standardized 40-pulse treatment. Efficacy was assessed as log reduction.

#### 2.3.3. Photorepair assessment.

UV-treated samples were divided for incubation for 3 hours at room temperature (~25°C) in either: a) Complete darkness or b) Visible light (under a standard cool white fluorescent lamp providing ~500 lux). Survival ratios were quantified after the incubation period to assess photoreactivation.

### 2.4. Statistical analysis

Results are expressed as mean ± standard deviation from three independent experiments (n = 3). One-way analysis of variance (ANOVA) followed by Tukey’s post-hoc test was used to analyze the effect of suspension depth. Student’s t-test was used for analyzing the significance of photorepair. D_10_-values (UV dose in mJ/cm² or number of pulses required for a 90% or 1-log₁₀ reduction) were calculated from the slope of the log-linear regression model fitted to the initial portion of the dose-response curves. Goodness-of-fit was assessed using the coefficient of determination (R²). A p-value of <0.05 was considered statistically significant.

## 3. Results

### 3.1. System spectral output and inactivation efficacy

**Fig 2:**
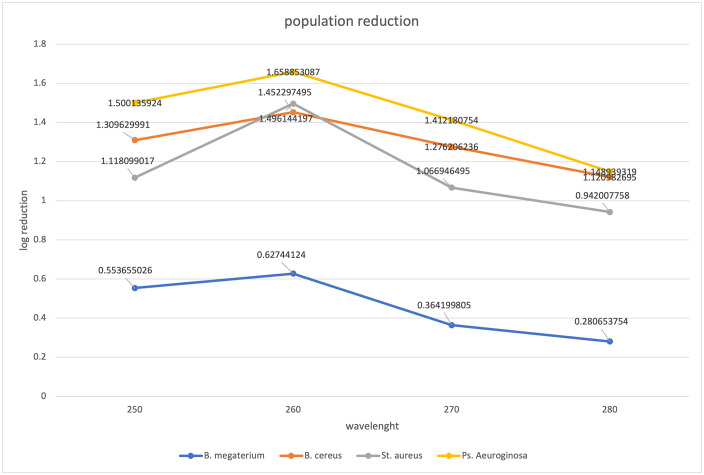
Emitted spectrum of the PUV system at different discharge voltages, showing strong output in the UVC region (Adapted from the manufacturer’s manual).

The xenon flash lamp produces a broad-spectrum output with significant peaks in the germicidal UVC range (Fig 2), which is responsible for the observed microbial inactivation.After exposure to 200 pulses (a cumulative dose of 108 mJ/cm²), the following log reductions were achieved: *P. aeruginosa*: 1.66 ± 0.15, *S. aureus*: 1.45 ± 0.12, *B. cereus*: 1.50 ± 0.11, and *B. megaterium* spores: 0.63 ± 0.09.

### 3.2. Inactivation kinetics

**Fig 3:**
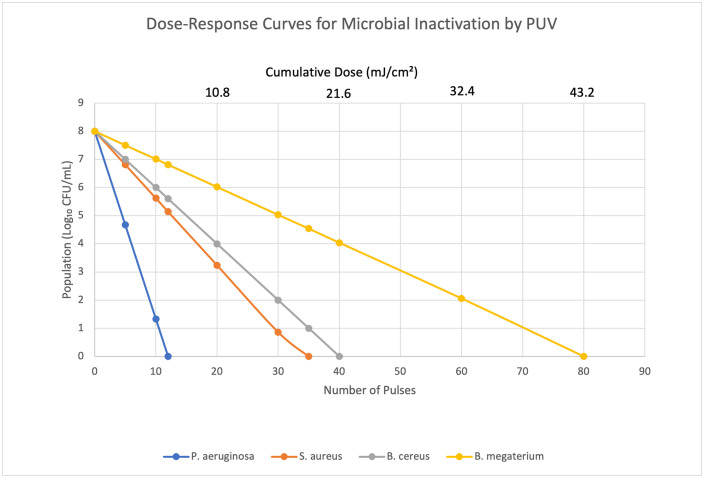
Dose-response curves for the inactivation of tested pathogens. The lines represent the log-linear inactivation model calculated from the D_10_-values presented.

Based on dose-response curves (Fig 3), D_10_-values were calculated and are presented in Table 1. *P. aeruginosa* was the most susceptible organism, while *B. megaterium* spores were the most resistant.

**Table 1:** Dose-response parameters for microbial inactivation by PUV.

Strain	Pulses for >6-log reduction	D_10_-value (Pulses)	D_10_-value (mJ/cm²)	R²
*P. aeruginosa*	12 ± 1	1.5	0.81	>0.99
*S. aureus*	35 ± 2	4.2	2.27	>0.98
*B. cereus*	40 ± 3	5	2.70	>0.98
*B. megaterium* spores	80 ± 4	10.1	5.45	>0.99

### 3.3. Depth-dependent efficacy

Increasing the volume (and thereby depth) of the suspension significantly reduced PUV effectiveness for *S. aureus* (p < 0.01), as detailed in Table 2.

**Table 2:** Effect of suspension volume on PUV efficiency (*S. aureus* after 40 pulses).

Volume (mL)	Log Reduction	Relative Efficacy (%)
20	6.8 ± 0.3	100
30	5.7 ± 0.4	84
40	4.2 ± 0.5	62
50	3.1 ± 0.6	46

### 3.4. Photorepair activity

Significant photoreactivation was observed in the two non-spore-forming species when exposed to visible light (Table 3). *P. aeruginosa* and *S. aureus* showed a statistically significant increase in survival compared to dark controls (p < 0.01).

**Table 3:** Photorepair activity under visible light (500 lux).

Strain	Light/Dark Survival Ratio
P. aeruginosa	1.32 ± 0.07*
S. aureus	1.28 ± 0.05*
B. cereus	1.05 ± 0.03
B. megaterium	1.02 ± 0.04

## 4. Discussion

This study systematically evaluated the efficacy of high-intensity pulsed ultraviolet light against key nosocomial pathogens, confirming its potent germicidal activity. Our main findings indicate that disinfection efficiency is critically dependent on the applied UV dose, pathogen type and physiology (vegetative vs. spore), and environmental conditions such as liquid depth.

Our dose-response analysis revealed significant differences in susceptibility among the tested organisms. *P. aeruginosa*, a Gram-negative bacterium, was the most sensitive (D_10_ = 0.81 mJ/cm²), which is consistent with previous research that often shows Gram-negative bacteria to be more susceptible to UV damage than Gram-positive bacteria, possibly due to differences in cell wall structure and thickness. The Gram-positive bacteria *S. aureus* and *B. cereus* required higher doses for inactivation.

The heightened resistance of *B. megaterium* spores (D_10_ = 5.45 mJ/cm²) is a well-documented phenomenon. This resistance is attributed to multiple factors, including the presence of α/β-type small acid-soluble spore proteins (SASPs) that alter DNA conformation, a dehydrated core, and a cortex layer containing dipicolinic acid that can absorb and scatter UV photons. These findings align with early work demonstrating the high resistance of *Bacillus* spores to PUV.

Furthermore, the inverse correlation between suspension depth and disinfection efficiency follows the Beer-Lambert law. Our data clearly show that increased liquid depth attenuates the photon flux reaching microorganisms throughout the sample, thereby decreasing the log reduction. This finding has practical implications for the disinfection of liquids,highlighting the need to ensure adequate mixing or thin-film exposure for effective treatment.

Finally, the observation of photorepair in *P. aeruginosa* and *S. aureus* underscores the importance of post-treatment conditions. The ability of bacteria to use visible light to enzymatically repair UV-induced DNA damage can lead to regrowth. This implies that for clinical applications, environments should be kept dark post-disinfection, or the applied UV dose must be high enough to cause irreparable damage beyond the capacity of cellular repair systems.

The clinical value of PUV lies in its rapid, chemical-free mechanism, ideal for high-risk zones [9] and heat-sensitive medical equipment [10,11]. The broad germicidal spectrum of pulsed xenon may also be more effective against a wider range of pathogens compared to traditional low-pressure mercury lamps [12]. For clinical application, careful consideration of safety,device standardization, and cost-effectiveness is crucial [13].

## 5. Study limitations

This study has several limitations. First, all experiments were conducted under optimized lab conditions using planktonic cells suspended in a clear, UV-transparent buffer (PBS). In real clinical settings, the presence of organic matter (e.g., serum, blood) can absorb UV photons, which would significantly reduce disinfection efficiency [14] and necessitate higher applied doses. Second, pathogens in hospitals often exist in biofilms, which can form a robust physical barrier against UV light. The efficacy of PUV against these complex structures was not assessed and requires further investigation. Third, potential shielding effects due to the complex geometries or surface irregularities of medical devices, which can lead to shadow zones, were not considered. This highlights the importance of device positioning and multiple treatment angles in practical applications. Finally, the long-term impact of repeated PUV exposure on material compatibility was not evaluated.

## 6. Conclusion

This study identifies three performance requirements for effective PUV disinfection: (1) Utilization of a broad-spectrum lamp with high output in the germicidal range, (2) Application of pathogen-specific dosing (e.g., a D_10_ dose of approximately 0.8 mJ/cm² for *Pseudomonas* vs. 5.5 mJ/cm² for *Bacillus* spores), and (3) Consideration of volume and depth for liquid disinfection to overcome UV attenuation. These parameters should be incorporated in the design and application of future hospital disinfection technologies.
